# Development of a Polyphenol Oxidase Biosensor from Jenipapo Fruit Extract (*Genipa americana* L.) and Determination of Phenolic Compounds in Textile Industrial Effluents

**DOI:** 10.3390/bios8020047

**Published:** 2018-05-15

**Authors:** Rafael Souza Antunes, Denes Ferraz, Luane Ferreira Garcia, Douglas Vieira Thomaz, Rafael Luque, Germán Sanz Lobón, Eric de Souza Gil, Flávio Marques Lopes

**Affiliations:** 1 Faculdade de Farmácia, Universidade Federal do Goiás (UFG), Rua 221 Esquina com a 5ª Avenida s/n, Setor Universitário, Goiânia-GO 74605-170, Brazil; rafael.antunes@panpharma.com.br or rafael.s.antunes@hotmail.com (R.S.A.); luane.fg@hotmail.com (L.F.G.); douglasvthomaz@gmail.com (D.V.T.); 2 Campus Henrique Santilo, BR-153, 3105, Universidade Estadual de Goiás, Fazenda Barreiro do Meio, Anápolis-GO 75132-903, Brazil; denes.bio@hotmail.com; 3 Departamento de Química Orgánica, Universidad de Cordoba, 14014 Cordoba, Spain; q62alsor@uco.es (R.L.); manger84@gmail.com (G.S.L.)

**Keywords:** *Genipa americana* L., polifenoloxidases, biosensor enzyme, phenolic compounds

## Abstract

In this work, an innovative polyphenol oxidase biosensor was developed from Jenipapo (*Genipa americana* L.) fruit and used to assess phenolic compounds in industrial effluent samples obtained from a textile industry located in Jaraguá-GO, Brasil. The biosensor was prepared and optimized according to: the proportion of crude vegetal extract, pH and overall voltammetric parameters for differential pulse voltammetry. The calibration curve presented a linear interval from 10 to 310 µM (r^2^ = 0.9982) and a limit of detection of 7 µM. Biosensor stability was evaluated throughout 15 days, and it exhibited 88.22% of the initial response. The amount of catechol standard recovered post analysis varied between 87.50% and 96.00%. Moreover, the biosensor was able to detect phenolic compounds in a real sample, and the results were in accordance with standard spectrophotometric assays. Therefore, the innovatively-designed biosensor hereby proposed is a promising tool for phenolic compound detection and quantification when environmental contaminants are concerned.

## 1. Introduction

The contamination of soil and water by industrial residues presents a huge threat to the environment due to their large wastewater output. In this context, the activity from food, cellulose, pharmaceutical and textile industries releases a myriad of pollutants, the highly stable structures of which may further hinder environmental remediation [1].

Amongst these pollutants, phenolic compounds are notorious, hence their toxicity towards human and animal organisms. These molecules are nonetheless highly versatile concerning industrial use and are employed in polyamide production by textile industries [2], as well as a plethora of other applications [3].

Concerning the presence of phenolic compounds in waterbodies, these molecules are prone to bond themselves to chlorate, nitrate, methyl and other alkyl moieties, thus disturbing environment physicochemical proprieties such as pH. Such disturbances, when associated with poor water treatment and human consumption, may henceforth lead to biological unbalances, which ultimately evolve to a variety of diseases [4,5].

Phenolic compounds are considered impactful even at minute concentrations and have been listed by both the United States Environmental Protection Agency and the European Union as toxic pollutants of high concern [2,6]. Hence, the harmful effects of such compounds and the development of new assessment tools and remediation strategies are noteworthy.

The analysis of phenolic compounds in environmental samples is mostly achieved through liquid chromatography [7], high performance liquid chromatography [8,9] and spectrophotometry [1,3]. However, such methods are expensive, unselective and mostly require the previous sample concentration. Therefore, the development of methods that allow fast, selective, low cost and practical contaminant assessment is of upmost importance [10].

Concerning phenolic pollutant analysis, biosensors are promising tools for their assessment, hence the enhanced selectivity of these devices, as well as fast analysis and low equipment cost. This kind of sensor is based on an enzymatic matrix, which provides process flexibility as the matrix organic portion can be altered according to the desired substrate under analysis [11,12,13].

Polyphenol oxidases (PPO) are a group of enzymes widely distributed in the plant kingdom and are responsible for phenol/quinone redox processes. These processes may be assessed by electrochemical methods such as voltammetry, which increases selectivity even further towards phenolic analytes. Furthermore, the ubiquitous distribution of PPO amongst plants allows different plant extracts to be used, and the accessibility provided by highly biodiverse biomes such as Cerrado further broadens the potential applications of biosensors [14,15,16].

Amongst potentially useful plants for biosensor development are Jenipapo (*Genipa americana* L.), a widely popular plant in Brazil whose folk use relies on its rich phenolic content. The presence of phenolic moieties among jenipapo secondary metabolites implies high PPO concentrations, which turns this fruit into an optimal source of crude vegetal extracts to be employed in biosensor production [1,14]

Therefore, the aim of this work was the development of a PPO-based biosensor from Jenipapo crude extract and employing it in the voltammetric assessment of phenolic compounds in industrial effluent samples obtained from a textile industry located in Jaraguá-Goiás.

## 2. Materials and Methods

### 2.1. Reagents and Solutions

All solutions and electrolytes were of analytical grade (Vetec Química Fina Ltd. (Rio de Janeiro, Brasil)). The water used was purified with a Millipore Milli-Q filter (Millipore S/A, (Molsheim, França)) and had an electrical conductivity of ≤0.1 µS cm^−1^. The catechol standard was acquired from Sigma-Aldrich (St. Louis, MO, USA). All standard solutions were prepared by dilution of 1 mM stock solutions, leading to a final concentration of 100 µM.

### 2.2. Vegetal Material and Crude Extract Preparation

Jenipapo fruits were collected from a single plant located in Anápolis-GO, Brasil, in January 2017 (geographic coordinates: 16°19′36″ S 48°57′10″ W). Ten fruits were collected, rinsed with water and stored in polyethylene recipients at 4 °C until analysis.

Crude vegetal extract was prepared by milling 30 g of fruits for 2 min in a food processor (Britania, Brazil) and adding therein 100 mL phosphate buffer solution (PBS) 0.05 M (pH 6.0). The solution was homogenized and filtrated on TNT fabric filter (nonwoven fabric), leading to a crude vegetal extract at 30% (Jenipapo enzymatic extract, JeEE) 0.01 M (pH 6.0). All procedures were conducted at room temperature (20 ± 2 °C).

### 2.3. PPO Enzymatic Activity

Spectrophotometry was used in order to evaluate PPO enzymatic activity. Therefore, 100 μL of JeEE were added to 3 mL of catechol solution 0.07 M in PBS media 0.05 M (pH 6.0), and absorbance at 420 nm was evaluated after 10 min using a spectrophotometer (Q798U2VS, Quimis Aparelhos Científicos Ltd., São Paulo, Brasil) [17]. PPO activity was expressed in U/mg protein.

The determination of total proteins was conducted according to Bradford [18], using bovine serum albumin (BSA) as the standard solution. Thereafter, 100 μL of JeEE and 5 mL Bradford reagent were added, and absorbance was evaluated at 595 nm. All assays were conducted at room temperature (20 ± 2 °C).

### 2.4. Biosensor Development

Carbon paste was prepared using graphite powder and mineral oil from Sigma-Aldrich (St. Louis, MO, USA). Enzyme immobilization from JeEE was performed by physical adsorption on carbon paste. The enzymatic extract was added directly to the graphite powder and then homogenized. Thereafter, it was subjected to drying at 16 ± 2 °C for a period of 30 min. The mineral oil, which acts as a binder, was then added, and the whole blend was further homogenized (Table 1).

**Figure 1:**
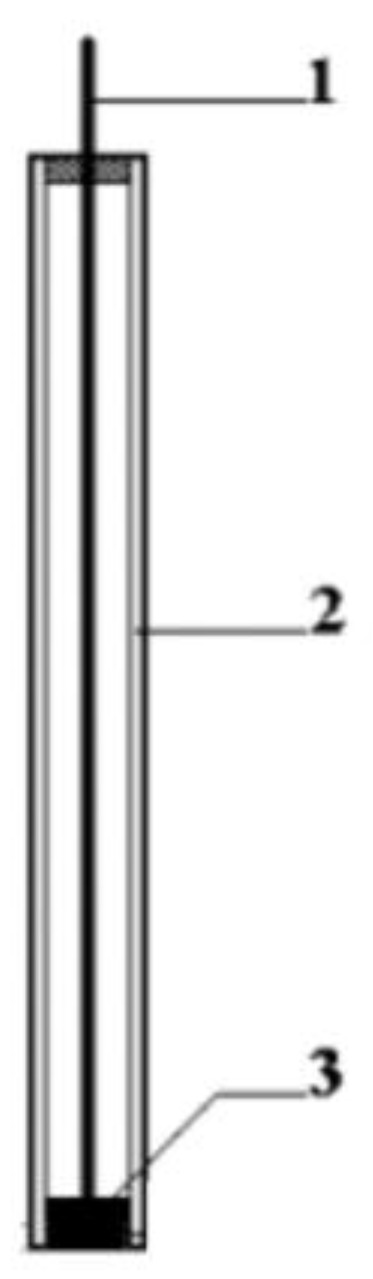
Cylindrical Teflon electrode (biosensor): (1) electric connection (copper wire); (2) Teflon lining; (3) carbon paste.

The prepared carbon pastes were prepared to fill a cylindrical Teflon electrode (Ø = 1 mm) as seen below (Figure 1).

The amount of enzymatic extract to be used was optimized by studying the capacity of four different JeEE biosensor proportions to detect 0.07 M catechol in PBS solution 0.05 M (pH = 6.0), and the tested proportions were: 50 µL (Carbon paste - Jenipapo 50 µL, CP-Jen50), 100 µL (CP-Jen100), 200 µL (CP-Jen200) and 300 µL (CP-Jen300), as shown in Table 1. The effect of pH in biosensor response towards catechol was evaluated for all systems using PBS 0.1 M, and pH was adjusted between 3.0 and 9.0.

### 2.5. Electrochemical Analysis and Stability Assay

Electrochemical analysis of oxidation products was determined by Differential Pulse Voltammetry (DPV) under the following experimental conditions: pulse amplitude 50 mV, pulse width 0.5 s, scan rate 10 mV s^−1^. Between each assay, the biosensor was subjected to 10 scans from 0 to 1.0 V at 100 mV s^−1^ to stabilize the signal. The measurements were carried out in a 1.0-mL electrochemical cell with a three-electrode system consisting of: a carbon paste electrode (CP, CP-Jen50, CP-Jen100, CP-Jen200 or CP-Jen300), a platinum wire and the Ag/AgCl/KCl 3 M, representing the working electrode, the counter electrode and the reference electrode, respectively. The voltammograms were background subtracted and baseline corrected. All data were analyzed using Origin 8^®^ software (OriginLab Corporation, Northampton, MA, USA). 

The storage stability test (shelf) was performed for 15 days. Five carbon pastes were prepared at the same concentrations of JeEE (CP-Jen100) and stored at 4 °C. After the following days, 1, 2, 5, 9 and 15, a sample (CP-Jen100) was recovered and allowed to warm at room temperature (20 ± 2 °C). The recovered carbon paste was used to fill the Teflon electrode (biosensor) and used to detect catechol in a 100 μM catechol solution.

### 2.6. Analytical Curve, Limit of Detection and Recovery Test

An analytical curve was constructed based on DPV assays conducted in solutions of 10 to 310 μM. LoD was defined as the smallest catechol concentration able to be detected using the chosen electrode, which therefore led to a LoD below 10 μM.

In order to study precision and accuracy, a recovery test was undertaken. Therefore, 20, 30, 40, 50 and 60 μM catechol solutions were added one at a time to a 100 μM catechol solution. The recovery was calculated as displayed below [6]:

$$\mathrm{Recovery}\;\left(\%\right)=\left(\frac{C1-C2}{C3}\right)\times 100$$

where C1 = final concentration; C2 = initial concentration; C3 = concentration of added solution.

### 2.7. Industrial Effluent Sample 

The effluent sample was obtained from a textile industry specialized in jeans textile coloration located in Jaraguá-GO, Brasil (geographic coordinates 15°45′25″ S 49°20′04″ W). The sample was collected before being subjected to any remediation procedure and stored in amber flasks until analysis. The samples were assessed using the proposed method to evaluate its viability towards real sample analysis.

### 2.8. Statistical Analysis

Statistical analysis was performed using the BioEstat program, Version 5.3. The statistic differences between groups were determined by the Student *t*-test. Statistical significance was considered as *p* > 0.05 (95%).

## 3. Results and Discussion

### 3.1. PPO Activity and JeEE Total Proteins

Preliminary studies concerning PPO enzymatic activity evidenced an activity of 593 U/mg proteins and an overall content of 1023 U/mg total proteins in 100 μL crude vegetal extract, which was maintained throughout a storage time greater than two months at 0 °C. The enzyme immobilization by adsorption was selected in this work due to its simplicity and easy execution [19,20].

The use of crude vegetal extracts on biosensors is a trend due to its analytical specificity, fast and reproducible analysis and excellent activity levels. Moreover, these enzymatic extracts have an extremely low cost, when compared to isolated enzymes. In this context, PPO-containing extracts are optimal for phenolic pollutant assessment [11,21,22].

Amongst PPOs, creolase and catecholase are responsible for the reversible conversion of *o*-diphenols to *o*-quinones. As these processes occur in potentials close to 0 V, their detection is feasible by carbon paste electrodes whose matrix presents adsorbed PPO enzymes [23,24].

The biological function of PPOs is strictly related to their capacity to reversibly bond to oxygen through a copper (Cu^2+^) active site, thus either oxidizing catechol to quinone or reducing quinone to catechol. These events are potential dependent and involve electron transfer, which is thereby detected in methods such as DPV [25,26]. 

**Scheme 1:**
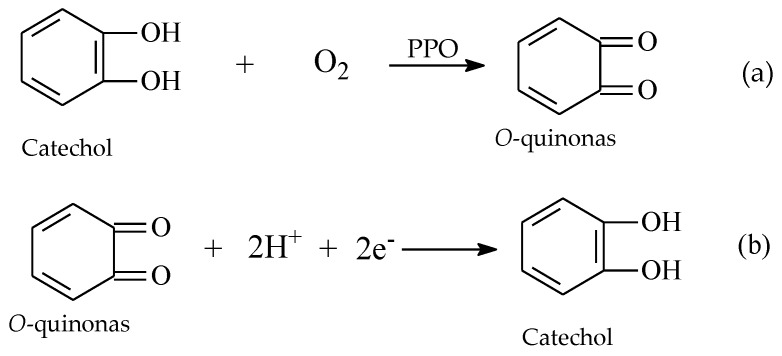
(**a**) PPO-mediated oxidative catalysis of catechol; (**b**) *o*-quinone reduction to catechol.

The oxidation of catechol can be assessed through an anodic scan, whereas the reduction of quinone to catechol is detected through a cathodic scan [25,26]. The aforementioned redox processes are represented below (Scheme 1).

### 3.2. Biosensor Optimization 

**Figure 2:**
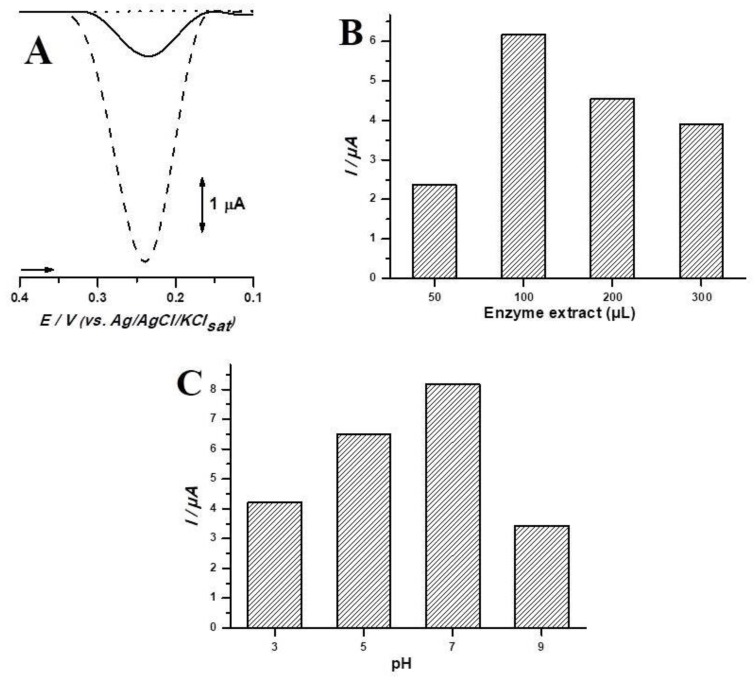
(**A**) Differential pulse voltamograms obtained with CP (▬) and CP-Jen100 (▪ ▪ ▪). Blank (•••). CP-Jen100 in PBS 0.1 M (pH 7.0). (**B**) Current peaks achieved through different crude vegetal extract proportions in the biosensor. (**C**) CP-Jen100 in PBS at different pH values. Analysis performed for a catechol solution of 0.07 mM in PBS 0.05 M (pH 6.0).

In order to investigate the benefits of the designed electrode and optimize its response towards industrial effluents’ analysis, a control test at different pHs was performed using an unmodified carbon paste electrode, as well as all different proportions of crude vegetal extract (Figure 2A–C).

The performed assay evidenced the highest peak amplitude belonging to JeEE, whose enzymatic matrix promotes specificity, as well as enhanced selectivity and sensibility (Figure 2A). The best response was obtained with 100 μL JeEE, which corresponds to an enzymatic activity of 593 U/mg proteins. It was clear that higher concentrations of crude vegetal extract lead to saturation, therefore hindering further detection and decreasing sensitivity (Figure 2B), and this event was previously reported in the literature [26]. The best pH was of 7.0 (Figure 2C). This value is in consonance with the literature, whose reports state better PPO activity at neutral pH values [14,15].

### 3.3. Analytical Curve for Catechol

**Figure 3:**
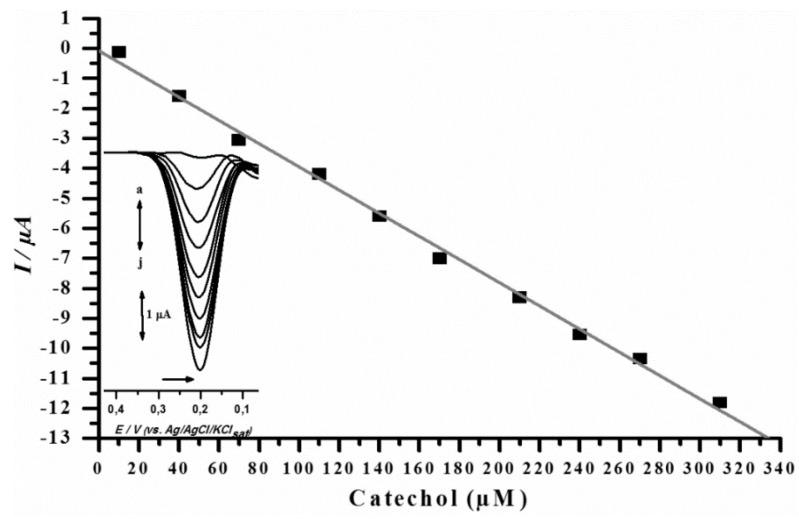
Analytical curve and DPVs obtained through CP-Jen100 at different catechol concentrations in PBS 0.1 M (pH 7.0).

The linearity of the proposed method was evidenced through an analytical curve. Results indicated that the method response was proportional to analyte concentration increment (Figure 3). Linearity was proven from catechol concentrations of 10 to 310 μM (r^2^ = 0.9982). Peak values varied from −0.368 to −12.7 μA, and curve equation corresponded to *I*/*µA* = −0.07958 − 0.03873 (catechol µM). Therefore, LoD was also determined, being 7 μM for CP-Jen100.

### 3.4. Stability Assay 

**Figure 4:**
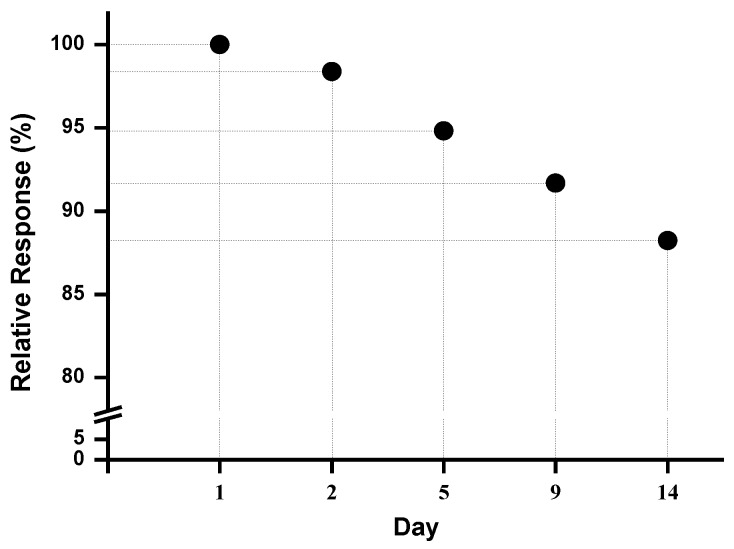
Relative response obtained according to the CP-Jen100 storage time. Analysis performed at 0.07 mM catechol solution in PBS 0.1 M (pH 7.0).

The CP-Jen100 stability was assessed during 15 days. At the end of the 15th day, 88.22% of the initial response was maintained towards the detection of catechol in a 100 μM solution (Figure 4). Therefore, enzyme immobilization through adsorption was efficient enough to resist lixiviation.

### 3.5. Recovery Assay 

The recovery assay was conducted in order to assess method precision and accuracy. Results indicated an accuracy of 87.50 to 96.00% (Table 2).

The recovery indexes were between 70% and 120%, which is in accordance with the literature concerning the analysis of textile industry phenolic contaminants [6], therefore indicating that the method was precise and accurate.

### 3.6. Analysis of Phenolic Contaminants in the Industrial Sample

Concerning phenolic contaminant analysis, tests are usually performed through spectrophotometry. Therefore, a comparison between a standard test and the hereby proposed test was needed in order to further prove its usefulness towards real sample assessment [6].

The obtained values for each assay, as well as the statistical significance are displayed below (Table 3).

The results show that no statistical difference can be attributed to the experimental values. Therefore, the method is reliable and reproductive. Moreover, a standard deviation of 0.84 was found for the biosensor, which is below that for spectrophotometry. Thus, the biosensor is more reliable than spectrophotometry analysis.

## 4. Conclusions

The designed biosensor was successfully employed to detect phenolic contaminants in a real sample from a textile industry, and no significant statistical difference between the described method and the standard spectrophotometric one was found. The new method presents nonetheless excellent recovery levels (70–120%). Furthermore, the biosensor was reliable, accurate, precise, robust and conformed to all analytical standards, being a reproducible tool to assess phenolic contaminants in industrial effluents.

## Figures and Tables

**Table 1:** PPO biosensor composition obtained from different Jenipapo crude extract proportions.

Biosensor *	Graphite Powder (mg)	Vegetal Extract (µL)	Mineral Oil (mg)
CP	100	-	30
CP-Jen50	100	50	30
CP-Jen100	100	100	30
CP-Jen200	100	200	30
CP-Jen300	100	300	30

* CP = carbon paste; Jen = Jenipapo.

**Table 2:** Recovery assay results for catechol standard using the jenipapo PPO biosensor.

Catechol (µM) ^1^	Catechol Added (µM) ^2^	Catechol Expected (µM) ^3^	Catechol Recovery (µM) ^4^	Catechol Recovery (%) ^5^
100	20	120	119 ± 0.93	95.00
100	30	130	127 ± 1.03	90.00
100	40	140	135 ± 0.77	87.50
100	50	150	148 ± 0.37	96.00
100	60	160	156 ± 1.22	93.33

^1^ Catechol standard solution. ^2^ Added catechol standard solution. ^3^ Recovered catechol standard solution. ^4,5^ Catechol recovery.

**Table 3:** Phenolic compounds detected in the industrial effluent sample by CP-Jen100 and spectrophotometry.

Method	Total Phenols (µM) (n = 4)	Student *t* Test
Spectrophotometry	238.90 ± 0.99	0.15
DPV using Jen100 biosensor	240.46 ± 0.84	
